# Circadian Effects on Attention and Working Memory in College Students With Attention Deficit and Hyperactivity Symptoms

**DOI:** 10.3389/fpsyg.2022.851502

**Published:** 2022-05-16

**Authors:** Lily Gabay, Pazia Miller, Nelly Alia-Klein, Monica P. Lewin

**Affiliations:** ^1^Department of Psychiatry, Icahn School of Medicine at Mount Sinai, New York, NY, United States; ^2^Department of Psychology, New York University, New York, NY, United States

**Keywords:** attention, chronotype, synchrony effect, working memory, ADHD

## Abstract

**Objective:**

Individuals with an evening chronotype prefer to sleep later at night, wake up later in the day and perform best later in the day as compared to individuals with morning chronotype. Thus, college students without ADHD symptoms with evening chronotypes show reduced cognitive performance in the morning relative to nighttime (i.e., desynchrony effect). In combination with symptoms presented in attention deficit hyperactivity disorder (ADHD), we predicted that having evening chronotype renders impairment in attention during the morning, when students require optimal performance, amplifying desynchrony.

**Method:**

Four hundred college students were surveyed for evening chronotype and symptoms of ADHD. Of those surveyed, 43 students with evening chronotype (19 with ADHD symptoms) performed laboratory attention tasks and were queried about fatigue during morning and evening sessions.

**Results:**

Students with ADHD symptoms demonstrated a greater decrement in sustained attentional vigilance when abstaining from stimulants and asked to perform cognitive tests at times misaligned with natural circadian rhythms in arousal compared to their non-ADHD counterparts with the same chronotype. While individuals with ADHD symptoms had slower reaction-times during sustained attention tasks in the morning session compared to those without symptoms, there was no significant group difference in working memory performance, even though both groups made more errors in the morning session compared to the evening session.

**Conclusion:**

These findings suggest that evening chronotype students with ADHD symptoms are at a greater disadvantage when having to perform sustained attention tasks at times that are not aligned to their circadian rhythm compared to their neuro-typical peers. The implications of this finding may be useful for the provision of disability accommodations to college age students with ADHD when they are expected to perform tasks requiring sustained attention at times misaligned with their circadian rhythms.

## Introduction

### Chronotype

Circadian rhythm is based on the sleep/wake cycle, which determines when it is time to sleep and when it is time to be awake. Different neurochemicals and hormones such as melatonin, cortisol, acetylcholine, and adenosine affect the sleep/wake cycle, causing it to rise and fall over the course of 24 h (Schmidt et al., 2007; Coogan and McGowan, 2017). As a result, sleepiness and alertness fluctuate at certain times of the day. Individual differences in circadian rhythm are largely based on chronotype, which refers to an individual’s biological preference in the timing of their sleep/wake cycle with respect to the 24-h day (Horne and Östberg, 1976). Differences in chronotype have a neurobiological basis with variations in blood pressure, hormone secretion, and core body temperature reflecting the time of day differences in arousal state (Schmidt et al., 2007; Lack et al., 2009). Chronotype varies from extreme morning type to extreme evening type, with an average of individuals falling in the middle as intermediate type. Individuals with an evening chronotype not only have later sleep and wake times than those with a morning type, but they also have a later timing of their circadian rhythms in core body temperature and cortisol levels, indicating later alertness (Schmidt et al., 2007; Kooij and Bijlenga, 2013; Randler et al., 2017). Many studies show individual variations in circadian arousal influence performance on tests of memory, attention, and decision making (May et al., 1993; May and Hasher, 1998; Schmidt et al., 2007; Valdez, 2019). These studies found that optimal cognitive functioning occurs when testing times are synchronized with individuals’ time of peak circadian arousal, which is referred to as the “synchrony effect.” While morningness is predominant in childhood, this tends to change during adolescence and throughout adulthood, with the highest prevalence of evening chronotype in college-aged individuals (Fischer et al., 2017; Randler et al., 2017). Thus, college students with evening chronotype experience a desynchrony between their “natural” times of alertness (i.e., evening) and the demands of college courses and testing which often happen in the mornings, at their time of least alertness.

### Attention Deficit/Hyperactivity Disorder

Evidence links evening chronotype and arousal instability with greater cognitive impairment in individuals with Attention-Deficit/Hyperactivity Disorder (ADHD), a neurological disorder marked by inattention and/or hyperactivity-impulsivity (American Psychiatric Association, 2013). Research shows that neural (Strauss et al., 2018) and behavioral (Mayer et al., 2016) correlates of arousal, alertness, and vigilance are impaired and more variable in young adults with ADHD compared to healthy individuals. ADHD is extremely prevalent in college age populations, with an estimate from 2012 of up to 8% of college students having a diagnosis of ADHD, and continually increasing diagnoses (Green and Rabiner, 2012). Students with ADHD exhibit considerable impairment with academic performance, including significantly lower grades and higher rates of school dropout in comparison to their peers (Green and Rabiner, 2012; Langberg et al., 2014).

### Attention Deficit Hyperactivity Disorder, Chronotype, and Sleep

There is a higher prevalence of evening chronotype among individuals with ADHD, with a rate of 73–78% in both children and adults (Bioulac et al., 2021); evening chronotype itself is associated with worse ADHD symptom severity (Coogan and McGowan, 2017). Past studies have also indicated that ADHD symptoms were found to be associated with a range of sleep disturbances such as higher subjective sleepiness, longer sleep latency, and later bedtime (Coogan et al., 2016). Given the high comorbidity of sleep problems in individuals with ADHD, circadian desynchrony may play a role in the progression of ADHD symptoms. Sleep problems are also shown to lead to worsening symptoms of inattention and hyperactivity individuals who do not necessarily have a formal diagnosis of ADHD (McGowan et al., 2016).

Furthermore, daytime sleepiness—which may occur when individuals with evening chronotype must adhere to a standard 8 AM to 6 PM class or work schedule—is associated with greater academic impairment for college students with ADHD (Langberg et al., 2014). While cognitive functioning will always fluctuate with circadian changes in arousal, individuals with ADHD may be less resilient to these circadian challenges due to prevailing deficits in arousal regulation. Although these studies suggest a possible trait-like profile that includes inter-relationships among chronotype, ADHD, and cognitive impairment, no studies have examined the relative magnitude of the acute effects of circadian desynchrony (i.e., lower performance on cognitive challenges when these are given in the morning) in individuals with ADHD who have an evening chronotype.

### Attention Deficit Hyperactivity Disorder, Working Memory, and Sustained Attention

Individuals with ADHD have been shown to have impairments in working memory (Willcutt et al., 2005; Mattfeld et al., 2016; Brydges et al., 2017) and impaired academic performance compared to individuals without ADHD (Biederman et al., 2004, 2006; Weyandt et al., 2013). Working memory function is implicated in higher-level cognitive processing, such as inhibitory control (Engle and Kane, 2004), reading comprehension (Daneman and Carpenter, 1980), and problem solving (Conway et al., 2003). Variations on working memory performance can be integral in determining academic functioning.

Adults with ADHD have also been shown to have impairments in attention, which is believed to be a hallmark of the disorder (American Psychiatric Association, 2013). Particularly, sustained attention is distinguished from other forms of attention because it requires individuals to maintain both arousal and activation to focus on one source of information for an extended period of time (Van der Meere and Sergeant, 1988). Prior research on sustained attention of adults with ADHD has shown deficits in sustained attention compared to controls (Epstein et al., 1998; Tucha et al., 2008; Avisar and Shalev, 2011) with decrements of sustained attention over time for individuals with ADHD (Tucha et al., 2017). Successfully being able to sustain attention is crucial for academic success for college students, given the challenges of lengthy lectures, extended and demanding exams, and independent study hours.

Here we address the question of whether individuals who report evening chronotype show impaired working memory and sustained attentional performance at desynchrony with their time of peak arousal and whether having symptoms of ADHD that can lead to a diagnosis further exacerbates these impairments. There are two phases of this study; in phase I, a large pool of participants were screened for chronotype and ADHD symptoms. In phase II, eligible participants were invited to participate in cognitive testing in the laboratory during morning and evening sessions.

## Phase I Method

### Participants

Undergraduate students were identified for recruitment from the New York University Subject Pool. Phase I consisted of a 15-min questionnaire administered through Qualtrics. Informed consent was obtained prior to completing the questionnaire and participants were compensated for their time with pay or extra credit toward a psychology course in which they were enrolled. Phase I respondents were excluded from analyses if (1) they did not complete the entirety of the prescreen questionnaire according to instructions; (2) they were not college-age (i.e., older than 24 years old); (3) they reported having a sleep disorder including Restless Leg Syndrome, Narcolepsy, Sleep Apnea, and sleep paralysis; or (4) they endorsed of a primary sleep disorder, or travel disruption (if they traveled out of the EST time zone in the 2 weeks prior to completing the survey). The final analysis for phase I included 363 respondents (270 women, 91 men, and 2 as other). Respondents ranged in age from 17 to 24 years old (*M* = 19.5, *SD* = 1.4). They self-identified as White/Eastern European (30%) Black or African American (9.1%), Hispanic or Latino (12.9%), Asian (39.7%), American Indian or Alaska Native (1.1%), Mixed Race (5.5%), and Other (1.7%).

### Materials

The questionnaire package for Phase I included 4 scales: the reduced Morningness-Eveningness Questionnaire (rMEQ) (Adan and Almirall, 1991), the ADHD-Adult Self Report Scale (ASRS) (Kessler et al., 2005), the Pittsburgh Sleep Quality Index (PSQI) (Buysse et al., 1989), and the Epworth Sleepiness Scale (ESS) (Johns, 1991). The rMEQ was used to evaluate participant chronotype (Adan and Almirall, 1991) was used to evaluate participant chronotype. According to the scoring criteria for chronotype (Adan and Almirall, 1991), we categorized individuals into three main groups based on their scores (evening type: < 10; intermediate type: 12–17; morning type: > 18). Next, we used the ASRS (Kessler et al., 2005) to screen for clinically significant levels of ADHD symptoms. Based on the ASRS scoring criteria, individuals who scored 4 points or above indicate current symptoms that are highly consistent with adults with ADHD and we refer to participants whose ASRS score reached the threshold for clinically significant levels of symptomatology (score > 4), as our with ADHD symptoms group (*n* = 98). Those who scored less than 4 are referred to as “without ADHD symptoms” (*n* = 265) (Kessler et al., 2005). The PSQI was used to assess overall sleep quality. On the PSQI, each dimension [(1) subjective sleep quality; (2) sleep latency; (3) sleep duration; (4) sleep efficiency; (5) sleep disturbance; (6) use of sleep medication; (7) daytime dysfunction] was scored and then summed to produce a global score ranging from 0 to 21, with higher global scores indicating worse sleep quality in individuals (Buysse et al., 1989). Finally, the ESS (Johns, 1991) was used to assess daytime sleepiness.

## Phase I Statistical Analyses

Data was analyzed in SPSS 25 (IBM Corp, 2019). Our analysis examined differences in sleep quality and daytime sleepiness measures (i.e., PSQI and ESS) between individuals with ASRS scores indicating ADHD clinical symptomatology (ASRS scores > 4) compared to healthy controls (individuals with ASRS scores that were not in the clinical range (ASRS scores < 4) using an independent sample *t*-tests (see Table 1). We used correlations to examine the relationship between ADHD symptoms, sleep quality, and daytime sleepiness and followed up with linear regressions to examine if ADHD symptomatology (ASRS) could predict sleep quality (PSQI) and daytime sleepiness (ESS). We also used chi-square tests of independence to assess whether individuals with ADHD clinical symptomatology (ASRS scores > 4) were more likely to indicate an evening chronotype (see Table 2).

**TABLE 1:** Sleep and ADHD criteria.

	Group							
	ADHD-criteria			Control				
Item	*N*	*M*	*SD*	*n*	*M*	*SD*	*Test*	*p*
Sleep quality	98	8.19	2.99	265	6.38	2.95	*t*(361) = −5.195	0.000
Day-time sleepiness	98	10.07	3.99	265	8.22	4.56	*t*(361) = −3.548	0.000

*An independent samples t-test reveals an indication of worse sleep quality and day-time sleepiness for individuals with ADHD. Significant at p < 0.001, two-tailed.*

**TABLE 2:** Self-reported chronotype by ADHD criteria.

	Chronotype category		
Group	Evening-type	Intermediate-type	Morning-type
Control	117 (44.2%)	128 (48.3%)	20 (7.5%)
**ADHD-criteria**	**58 (59.2%)**	**36 (36.7%)**	**4 (4.1%)**

*Chi square reveals a significant relationship between ASRS scores in the clinical range and evening chronotype [χ^2^ (2) = 6.77, p < 0.05].*

*The bold means that the result was significant.*

## Phase II Method

### Participants

Phase II of the study consisted of a subset of respondents from phase I. Respondents from phase I who met criteria for phase II were then emailed to alert them to their eligibility for a face-to-face phase II experiment at the NYU laboratory. Only respondents whose composite rMEQ score indicated an evening chronotype (score < 12) were eligible to progress to phase II of the study (*n* = 169). Participants were further excluded from participating in phase II if they traveled out of the EST time zone in the 2 weeks prior to completing the survey. By excluding participants who indicated any of the above, we were able control for any confounding effect of these factors on arousal variability or temporary changes in chronotype. Participants who reported taking stimulant medications were instructed not to modify their typical medication schedule, but instead to schedule their test sessions on a day they would normally abstain from any prescribed stimulant medications (i.e., a “medication holiday”). Written informed consent was obtained for these participants who qualified and chose to enroll (*n* = 43) and all procedures were approved and overseen by the New York University Institutional Review Board. Each in-person test session took approximately 45 min, totaling up to 2 h for the complete study. Participants were compensated for their time with cash or extra credit toward a psychology course in which they were enrolled. In this phase, 43 participants (28 women and 15 men) ranging in age between 18 and 33 (*M* = 20.4, *SD* = 2.6) were tested in person. Participants self-identified as White/Eastern European (30.2%) Black or African American (14%), Hispanic or Latino (11.6%) and Asian (44.2%). Of the 43 participants, the group without ADHD symptoms consisted of 16 women and 8 men (*M*_*age*_ = 21.1, *SD* = 3.2); the group with ADHD symptoms consisted of 12 women and 7 men, (*M*_*age*_ = 19.6, *SD* = 1.4).

### Materials

We used a python-based computerized Sustained Attention to Response Task (SART) in order to assess lapses of sustained attention, processing speed, and error rates in this performance based task (Robertson et al., 1997). We used the total error score on the SART as an objective measure of sleepiness, indicating vigilance impairment (Robertson et al., 1997; Van Schie et al., 2012). In the SART task, participants were shown a number from 1 to 9 and were instructed to press a key each time a number was shown except for the number 3. We also used a variation of the N-back task (Owen et al., 2005; Kane et al., 2007) to assess working memory performance. N-back performance was assessed on three different N-back loads: 1-back, 2-back and 3-back tasks, in which participants were asked to respond to numbers that were presented one, two, and three trials earlier. At each test session, participants were administered the Stanford Sleepiness Scale (SSS) (Hoddes et al., 1973) and a “Sleep Diary” questionnaire, where we asked participants how many hours they slept the night before, if they used any sleep aids, or if they had used any substances (alcohol, caffeine, or stimulant medications) the night prior or morning of the study. Participants who reported (a) < 5 h of sleep, (b) use of any sleep aiding substances, or (c) alcohol, drug, or caffeine consumption, were permitted to complete the first session but later excluded from our analyses. Two participants were excluded from the analyses due to self-reported caffeine consumption prior to the study.

### Procedure

These procedures were followed for each participant in phase II: Morning session started upon arrival to the lab at 8:00 AM, informed consent was obtained, followed by the “Sleep Diary” questionnaire. Following this, we administered the computerized N-back task and SART. Evening session started upon returning to the lab at 9:00 PM, where we administered a second “Sleep Diary” questionnaire asking participants if they have napped or used any substances (alcohol, caffeine, or stimulant medications) since Session 1; if so, they were later excluded from analyses. Participants were again administered the SSS to indicate their current level of subjective sleepiness. Participants then completed the same version of the computerized N-back task and SART as Session 1.

## Phase II Statistical Analyses

**FIGURE 1:**
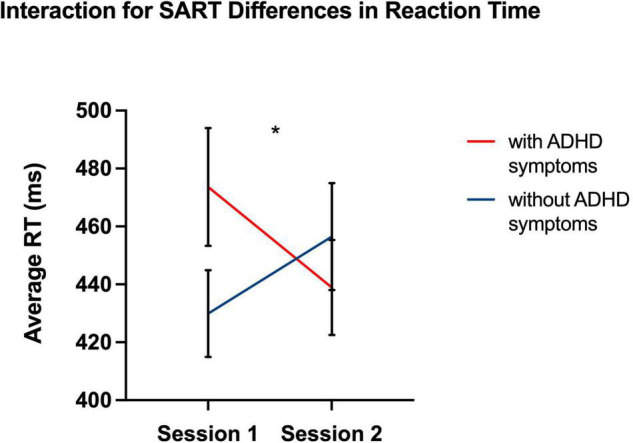
Graph showing a significant interaction effect for Session × Group. As a result, response latency for individuals with ADHD was greater in Session 1 than in Session 2 than for neuro-typical individuals. *p < 0.05, two-tailed.

**FIGURE 2:**
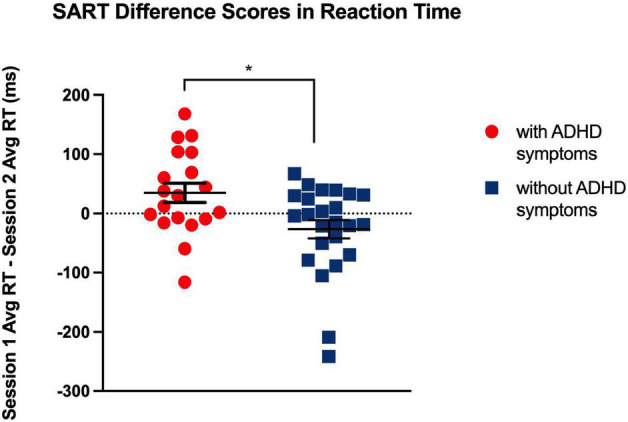
Significant differences in response latency, at *p* < 0.05, between Session 1 and 2 indicate a greater magnitude of the synchrony effect for individuals with ADHD compared to neuro-typical individuals. *p < 0.05, two-tailed.

**FIGURE 3:**
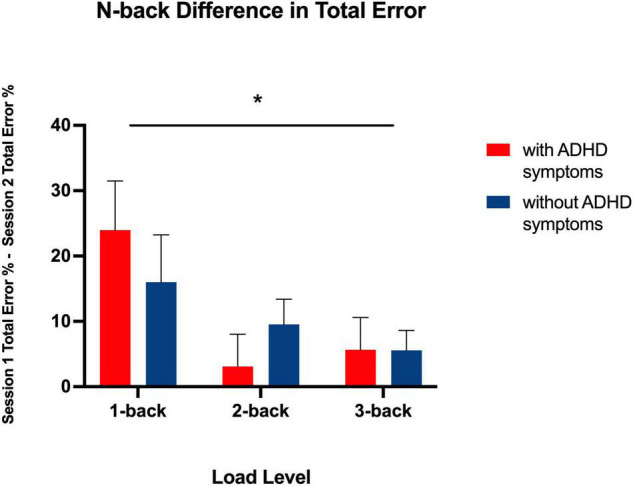
Bar graph showing total error difference scores between Session 1 and 2 for each N-back level in both groups. * indicates a significant main effect of N-back level, *p* < 0.05, two-tailed. This shows a chronotype dependent effect on performance for all levels.

**FIGURE 4:**
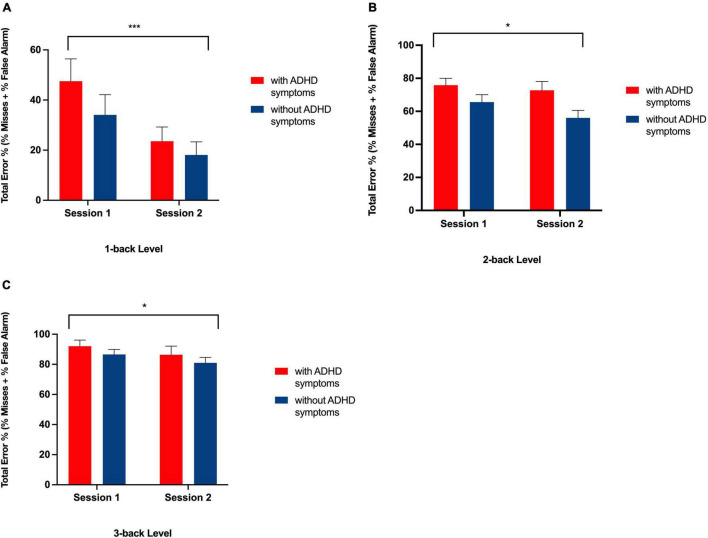
Bar graph showing total error rates on the N-back task at Session 1 and Session 2 for each group at the 1-back **(A)**, 2-back **(B)**, and 3-back **(C)** level. ***Indicates a significant main effect of session, *p* < 0.01, * indicates a significant main effect of session, *p* < 0.05 with more errors being made at Session 1 than at Session 2 in both groups.

**FIGURE 5:**
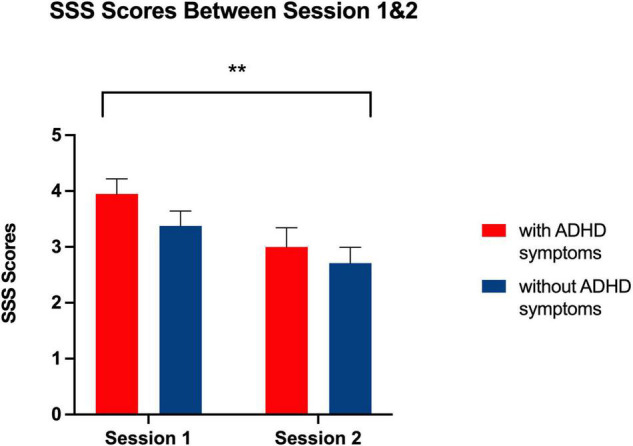
Bar graph showing Stanford Sleepiness Scale Ratings at Session 1 and 2 for each group. **Indicates a significant main effect of session, *p* < 0.01, with higher ratings of self-reported sleepiness being reported at Session 1 than Session 2 related to all participants’ evening chronotype.

We tested the assumption that individuals with evening chronotype would demonstrate a time-of-day dependent change in both subjective sleepiness and cognitive performance. Total error rate for the SART task was calculated by summing the rate of omission errors (misses) and commission errors (false alarms). Omission errors occurred when no key was pressed in cases where a key should have been pressed (i.e., after any number but 3); commission errors occurred when a key was pressed when no key should be pressed (i.e., after a 3). Reaction time for each response was collected to assess processing speed (Robertson et al., 1997), and the average reaction time was calculated at each session. Sleepiness (SSS) scores ranged from 1 to 7, with lower scores indicating higher arousal and higher scores indicating greater subjective sleepiness. Using GraphPad Prism version 8.10 software, we conducted a 2 (with ADHD symptoms vs. without ADHD symptoms) × 2 (evening vs. morning) repeated measures ANOVA for measures of working memory and sustained attention, and an independent sample *t*-test to measure differences in sustained attention and sleepiness by group (see Tables 3–5 and Figures 1–5). We used a series of ANOVAs due to limited sample size and Bonferroni corrections for multiple comparisons. Differences in scores were calculated as Session 1 (morning session)—Session 2 (evening session) in order to control for individual variations in cognitive performance while still measuring the degree of change across sessions. Therefore, a positive difference score for total error rate would indicate an individual’s performance in the morning was impaired relative to their performance on the same task later that evening. We predicted that performance accuracy would be worse in the morning (Session 1) than in the evening (Session 2) for both the group with ADHD symptoms and the without ADHD symptoms for each task. As our study only included participants with evening chronotype, we predicted average sleepiness SSS scores would be greater in the morning (Session 1) than in the evening (Session 2) across both groups. A 2 (with ADHD symptoms vs. without ADHD symptoms) × 2 (Session 1 vs. Session 2) was conducted for this purpose (see Figure 1). ANCOVA was performed to assess the difference of the slopes between performance on the SART and N-back for each group.

**TABLE 3:** Sustained attention to response task analysis of variance.

	With ADHD symptoms (*n* = 19)		Without ADHD symptoms (*n* = 24)		Main effect of Group *F*(1, 41)	Main effect of Session *F*(1, 41)	Session × Group Interaction *F*(1, 41)
	*M*	*SD*	*M*	*SD*			
*a.* Total error score at each session	59.68^1^ 59.58^2^	14.77 17.82	62.04^1^ 55.26^2^	17.29^1^ 20.51^2^	0.04	1.78	1.67
***b.* Average reaction time (ms) at each session**	**473.6^1^ 438.9^2^**	**88.63** **71.43**	**429.9^1^ 456.5^2^**	**73.35^1^** **90.36^2^**	**0.34**	**0.13**	7.26*

Significant at *p < 0.05, two-tailed.

*ANOVA reveals a significant Group × Session interaction for SART reaction time. The mean total error score and average reaction time, in milliseconds, is shown at session at for each group.*

*^1^Session 1, ^2^Session 2.*

*The bold means that the result was significant.*

**TABLE 4:** Sustained attention to response *t*-test.

	With ADHD symptoms (*n* = 19)		Without ADHD symptoms (*n* = 24)		*t*(40)	*p*
	*M*	*SD*	*M*	*SD*		
*a.* Total error difference score	0.10	16.04	6.79	17.42	1.29	0.203
***b.* Average reaction time difference score**	**515.43**	**99.56**	**483.31**	**98.97**	2.70*	** < 0.05**

Significant at *p < 0.05, two-tailed.

*T-test reveals a significant difference in reaction time (RT) for the group with ADHD symptoms.*

*a, the mean total error difference score and average RT difference scores for each group. Total error difference scores were calculated by subtracting the total error rate (% of misses + % of false alarms) in Session 1 from the total error rate in Session 2.*

*b, average RT difference scores were calculated by subtracting the average reaction time for Session 1 from Session 2 for each individual.*

*The bold means that the result was significant.*

**TABLE 5:** N-back total error scores at each session.

	With ADHD symptoms (*n* = 19)		Without ADHD symptoms (*n* = 24)		Main effect of Group *F*(1, 41)	Main effect of Session *F*(1, 41)	Session × Group Interaction *F*(1, 41)
	*M*	*SD*	*M*	*SD*			
1-back load	105.51^1^ 77.05^2^	38.81^1^ 24.85^2^	34.08^1^ 18.07^2^	39.52^1^ 25.75^2^	1.19	14.43***	0.57
2-back load	107.10^1^ 115.32^2^	18.28^1^ 23.31^2^	65.58^1^ 56.04^2^	22.10^1^ 22.25^2^	5.23*	4.20*	1.09
3-back load	121.98^1^ 113.91^2^	17.61^1^ 24.85^2^	86.56^1^ 80.98^2^	16.34^1^ 17.91^2^	1.11	4.09*	0.0003

*ANOVA revealing a significant main effect of session at each N-back load.*

*One participant in the group with ADHD symptoms was excluded from the 1-back analysis, n = 18.*

***Indicates a significant main effect, p < 0.001, two-tailed.

*Indicates a significant main effect, p < 0.05, two-tailed.

*^1^, session 1, ^2^, session 2.*

## Phase I Results

### Correlation of Sleep Measures

PSQI and ESS scores were correlated for all participants [*r*^2^ (363) = 0.243 *p* < 0.001, two-tailed], indicating a significant positive relationship between global sleep quality and daytime sleepiness. This remained true for individuals who met criteria for ADHD (ASRS score > 4) [*r*^2^ (98) = 0.366 *p* < 0.01, two-tailed] and those who did not meet ADHD criteria [*r*^2^ (265) = 0.209 *p* < 0.01, two tailed].

### Attention Deficit Hyperactivity Disorder Clinical Symptomatology and Sleep Measures

Individuals who met criteria for an ADHD diagnosis had indicating worse sleep [*t* (361) = −5.20, *p* < 0.001, two-tailed] and higher levels of daytime sleepiness, than those who did not meet criteria [*t* (361) = −3.55, *p* < 0.001, two-tailed] (see Table 1). Similarly, there was a significant correlation between ADHD symptom scores and worse sleep [*r*^2^ (363) = 0.393, *p* < 0.001, two-tailed], and daytime sleepiness [*r*^2^ (363) = 0.283, *p* < 0.001, two-tailed]. A significant linear regression further shows ADHD symptom scores as a predictor of impaired sleep [*F* (1, 361) = 66.02, *p* < 0.0001] and daytime sleepiness [*F* (1, 361) = 22.69, *p* < 0.0001].

### Attention Deficit Hyperactivity Disorder Clinical Symptomatology and Chronotype

There was a significant relationship between ADHD symptom scores in the clinical range and evening chronotype [χ^2^ (2, 363) = 6.77, *p* < 0.05], suggesting a higher prevalence of evening chronotype in individuals who may meet criteria for a clinical diagnosis of ADHD (see Table 2).

## Phase II Results

### Sustained Attention

Despite null differences in sleepiness, during task performance, there was increased reaction-time during the sustained attention task in the ADHD symptoms group compared to the group without ADHD symptoms during the morning (Session 1) as compared to evening session (Session 2) [*F* (1, 41) = 7.26, *p* = 0.01] (see Table 3 and Figure 1), indicating a slower response. Thus, that difference in reaction-time between the morning session and the evening session was significantly greater in the ADHD symptoms group (*M* = 515.43, *SD* = 99.56) than in the group without symptoms (*M* = 483.31, *SD* = 98.97) *t* (41) = 2.70, *p* < 0.05 (see Table 4 and Figure 2).

### Working Memory

As expected, there was a significant main effect for N-back level [*F* (2, 82) = 5.31, *p* < 0.01], such that in both the ADHD symptoms and without symptoms groups, the difference in the total amount of errors increased as cognitive load increased (from 1-back, to 2-back, to 3-back) (see Table 5 and Figure 3). Each N-back level revealed a significant main effect of session, such that both groups performed worse in the morning session (see Figure 4). However, there was no significant main effect of Group (with ADHD symptoms vs. without ADHD symptoms), [*F* (1, 41) = 0.01, *p* = 0.918], and no significant interaction between Group and N-back Level [*F* (2, 82) = 1.05, *p* = 0.354] (see Table 5).

### Subjective Sleepiness

As expected for their evening chronotype, participants in Phase II reported greater subjective sleepiness during the morning session conducted at 8:00 AM compared to the evening test session conducted at 9:00 PM [*F* (1, 41) = 0.958, *p* < 0.01] (see Figure 5). However, there was no main effect of group [*F* (1, 41) = 1.80, *p* = 0.187] and no session by group interaction [*F* (1, 41) = 0.29, *p* = 0.593] that would indicate a greater degree of sleepiness at any given session for individuals with ADHD symptoms as compared to those without ADHD symptoms.

### Objective Sleepiness

Although participants reported greater subjective sleepiness in the morning session as compared to the evening session, there was no significant difference between the groups in SART total error score by session [*F*(1, 41) = 1.67, *p* = 0.203], indicating no difference in impaired vigilance by time of day between groups. Additionally, there were no differences in total error score by session [*F* (1, 41) = 1.78, *p* = 0.190] and by Group [*F* (1, 41) = 0.04, *p* = 0.840] (see Table 3). However, those without ADHD symptoms trended toward greater impaired vigilance in the evening session compared to the morning session, [*t* (23) = 1.91, *p* = 0.07, two-tailed], a trend that was not present in individuals with ADHD symptoms. Overall, there was no significant difference in objective sleepiness for individuals with ADHD symptoms compared to those without ADHD symptoms.

## Discussion

Our study aimed to measure the relative impact of circadian desynchrony effects on working memory and sustained attention between college students with and without ADHD symptomatology. We expected to find a greater decrement in both working memory and sustained attention for students with ADHD symptoms compared to those without during the morning session compared to evening. Indeed, there was increased impairment for those with ADHD symptoms in cognitive functioning. In our study, participants with ADHD symptoms demonstrated a significantly greater reaction time on sustained attention tasks when assessed at times desynchronous to their circadian rhythms as compared to those without ADHD symptoms—a finding that has not been studied or observed elsewhere. Although this group difference in synchrony effect was not observed for total errors made on sustained attention task, poorer sustained attention during desynchronous times suggests that ADHD symptoms are exacerbated during desynchronous cognitive performance. Even though there were no significant differences in total errors on the sustained attention task or on the working memory task, differences in reaction time indicate decremented sustained attention. Poorer sustained attention may result in a number of increased academic challenges, such as limited endurance when completing complex cognitive tasks and overall impaired academic performance. Therefore, our results suggest that individuals with ADHD symptoms are at an increased decrement when performing tasks desynchronous with their circadian rhythms compared to their peers. Existing studies have found that individuals with ADHD demonstrate poorer sustained attention due to sleepiness compared to neuro-typical controls (Helfer et al., 2020), but no studies have examined the impact of performance desynchrony on sustained attention performance. The implications of this finding may be useful for the provision of disability accommodations to college age students with ADHD when they are expected to perform tasks requiring sustained attention at times misaligned with their circadian rhythms.

We found that overall both groups showed improved working memory at times in synchrony with their circadian rhythms, especially as the cognitive load increases; a finding which is supported by existing literature (May and Hasher, 1998; Yoon et al., 1998; May, 1999; Ramírez et al., 2006; Goldstein et al., 2007; Rowe et al., 2009; Hahn et al., 2012; RamÍrez et al., 2012; Lehmann et al., 2013). However, there was no interaction between ADHD symptoms and session time in working memory performance, suggesting that chronotype outweighs the presence of ADHD symptoms in creating a decrement in working memory performance. While our findings suggest individuals with ADHD symptoms are not at a relative disadvantage for working memory compared to their neuro-typical peers, chronotype should be taken into consideration when assessing working memory capacity, even at low levels of difficulty. Although previous studies demonstrated a synchrony effect on n-back performance only at high-load levels (2-back and 3-back) (Schmidt et al., 2007), our results introduce novel evidence for the synchrony effect at low working memory load levels (1-back). Since evening-chronotype is over-represented in college-age individuals, our results suggest that class and exam times be shifted to later in the day in order to capture students’ academic and cognitive ability more accurately.

Finally, we found higher reporting of subjective sleepiness in the morning than in the evening for all students, as expected for students with evening chronotypes. However, we did not find any decrement in the objective measure of impaired vigilance by time of day for both groups. This finding suggests that feeling sleepy and attentional impairments are two different but related mechanisms, such that feeling sleepy does not necessarily result in impaired attentional vigilance (Fronczek et al., 2006; van der Heide et al., 2015). In addition to our main findings on the relative effects of circadian desynchrony on individuals with ADHD symptoms, our study supports the literature on sleep problems and ADHD. Here, Phase I data indicated that individuals with ADHD symptoms have a higher prevalence of evening chronotype than their neuro-typical peers, an effect which has been previously shown (Rybak et al., 2007; Baird et al., 2012). Our study also revealed college-age individuals with ADHD symptoms experience a greater degree of daytime sleepiness (Chiang et al., 2010) and worse subjective sleep quality than their peers, consistent with existing literature (Boonstra et al., 2007; Rybak et al., 2007; Sobanski et al., 2008). Our study also investigates the potential interaction between daytime sleepiness and sleep quality in ADHD-symptomatic adults, and found that ADHD symptomatology was predictive of both poorer sleep quality and daytime sleepiness, consistent with the theory that core symptoms of ADHD may be derivative of sleep problems (Corkum et al., 1998) and changes in the circadian system (Rybak et al., 2007; Baird et al., 2012).

Several limitations to the work described should be noted. First, because of our extensive inclusion/exclusion criteria, we were only able to recruit and include a limited number of participants in our Phase II analyses (without ADHD symptoms group: 18 women, 6 men; with ADHD symptoms group: 12 women, 7 men). Second, it is possible that there was a rebound effect for individuals in the ADHD symptoms group who did not take stimulant medication that day, whereas these symptoms would otherwise be controlled. Skipping a stimulant dose may reduce procedural memory performance for individuals on that day (Fox et al., 2014), but that effect may only be present when individuals are un-medicated. Due to this design decision, however, the study is able to demonstrate the naturalistic effects of ADHD symptomatology on desynchronous working memory performance. Another limitation of note is that the duration of the cognitive tasks that participants performed may not be representative of real-life circumstances. Here, each task lasted about 6 min, and daily cognitive tasks may require more or less extended periods of focus. Additionally, all participants were administered the morning test first and evening test after, so it is possible that the significant improvements in reaction time are due to test-retest learning effects. Finally, a limitation that has been similarly addressed in previous studies is the method used to assign participants to the ADHD symptoms group (Green and Rabiner, 2012). Our methods relied on self-reported diagnostic status and/or threshold symptoms based on the ASRS (8 participants reported having a diagnosis of ADHD and 11 participants indicated ADHD symptoms in the clinical range for a diagnosis), rather than using in-person assessment by a study clinician for enhanced accuracy. Future studies should aim to correct these limitations.

Importantly, our work points to a need for additional research assessing the potential interaction of chronotype and time of day on other domains of cognitive functioning. Another high-potential candidate for an ADHD-desynchrony effect may be response inhibition, a major feature of ADHD-related cognitive impairment (Epstein et al., 2001; Wodka et al., 2007). Additionally, future research should be extended to include additional measures of sustained attention and reaction time, such as the Psychomotor Vigilance Test (PVT) and driving simulators (Dinges and Powell, 1985; Schmidt et al., 2007) in order to assess the possibility of greater relative impairment in individuals with ADHD similar to the effects found here. Such findings may have implications for disability accommodations as well as other safety considerations, for individuals with ADHD at times desynchronous with their circadian rhythm—which may prevent the frequency of critical safety issues such as workplace accidents and errors, and automobile accidents.

Overall, our results demonstrate the importance of observing time of day effects on performance and its effects on individuals with ADHD symptoms, as it informs potential interventions to support the overall success of college students and others suffering from the disorder.

## Data Availability Statement

The raw data supporting the conclusions of this article will be made available by the authors, without undue reservation.

## Ethics Statement

The studies involving human participants were reviewed and approved by New York University Institutional Review Board. The patients/participants provided their written informed consent to participate in this study.

## Author Contributions

LG and MPL contributed to the design and implementation of the research, to the analysis of the results, and to the writing of the manuscript. NA-K and PM contributed to the analysis of the results and writing of the manuscript. All authors contributed to the article and approved the submitted version.

## Conflict of Interest

The authors declare that the research was conducted in the absence of any commercial or financial relationships that could be construed as a potential conflict of interest.

## Publisher’s Note

All claims expressed in this article are solely those of the authors and do not necessarily represent those of their affiliated organizations, or those of the publisher, the editors and the reviewers. Any product that may be evaluated in this article, or claim that may be made by its manufacturer, is not guaranteed or endorsed by the publisher.
